# Advances in Breeding for Mixed Cropping – Incomplete Factorials and the Producer/Associate Concept

**DOI:** 10.3389/fpls.2020.620400

**Published:** 2021-01-11

**Authors:** Benedikt Haug, Monika M. Messmer, Jérôme Enjalbert, Isabelle Goldringer, Emma Forst, Timothée Flutre, Tristan Mary-Huard, Pierre Hohmann

**Affiliations:** ^1^Department of Crop Sciences, Research Institute of Organic Agriculture FiBL, Frick, Switzerland; ^2^Université Paris-Saclay, INRAE, CNRS, AgroParisTech, GQE – Le Moulon, Gif-sur-Yvette, France; ^3^Dipartimento di Scienze Agrarie, Alimentari ed Ambientali (D3A), Università Politecnica delle Marche, Ancona, Italy; ^4^UMR MIA-Paris, AgroParisTech, INRA, Université Paris-Saclay, Paris, France

**Keywords:** mixed cropping, intercropping, breeding, general mixing ability, producer/associate concept, incomplete factorial design, biological interaction, simulations

## Abstract

Mixed cropping has been suggested as a resource-efficient approach to meet high produce demands while maintaining biodiversity and minimizing environmental impact. Current breeding programs do not select for enhanced general mixing ability (GMA) and neglect biological interactions within species mixtures. Clear concepts and efficient experimental designs, adapted to breeding for mixed cropping and encoded into appropriate statistical models, are lacking. Thus, a model framework for GMA and SMA (specific mixing ability) was established. Results of a simulation study showed that an incomplete factorial design combines advantages of two commonly used full factorials, and enables to estimate GMA, SMA, and their variances in a resource-efficient way. This model was extended to the Producer (Pr) and Associate (As) concept to exploit additional information based on fraction yields. It was shown that the Pr/As concept allows to characterize genotypes for their contribution to total mixture yield, and, when relating to plant traits, allows to describe biological interaction functions (BIF) in a mixed crop. Incomplete factorial designs show the potential to drastically improve genetic gain by testing an increased number of genotypes using the same amount of resources. The Pr/As concept can further be employed to maximize GMA in an informed and efficient way. The BIF of a trait can be used to optimize species ratios at harvest as well as to extend our understanding of competitive and facilitative interactions in a mixed plant community. This study provides an integrative methodological framework to promote breeding for mixed cropping.

## Introduction

Climate change, such as rising global temperatures and climatic volatility are predicted to jeopardize future agricultural productivity (Rahmstorf and Coumou, 2011). The current strategies to produce stable and high yields, e.g., by the application of mineral fertilizer, are of limited future use since they themselves are a contributor to these changing climatic parameters (Thompson et al., 2019). Thus, alternative approaches to achieve high and stable yields while maintaining biodiversity and minimizing environmental impact have to be developed. Mixed cropping is the simultaneous cultivation of two or more crops on the same field. Especially legume/non-legume species mixtures have been proposed to achieve a higher per area production and profitability and higher yield stability with less or no external inputs (Bedoussac et al., 2015; Raseduzzaman and Jensen, 2017; Wendling et al., 2017; Viguier et al., 2018). Good pairs of complementary species have already been identified, such as combinations of corn (*Zea mays* L.) with cowpea (*Vigna unguiculata* L., Ofori and Stern, 1986), with common bean (*Phaseolus vulgaris* L., Hoppe, 2016; Starke, 2018), and with faba bean (*Vicia faba* L., Li et al., 2020), as well as small grain cereals such as barley (*Hordeum vulgare* L., Hauggaard-Nielsen et al., 2001) with pea (*Pisum sativum* L.) or wheat (*Triticum aestivum* L.) with faba bean (Agegnehu et al., 2006) or with lentil (*Lens culinaris* MEDIK., Viguier et al., 2018).

The choice of the genotypes best suited to mixing within each species is not straight-forward, and a robust strategy for evaluating and identifying the right mixing partners from among a large number of candidates is essential to selecting the components of mixtures and improving the mixing ability of each species. Selection efficiency for mixed cropping yield under pure stand has been reported to be moderate or low highlighting the value dedicated breeding efforts for mixed cropping (de Oliveira Zimmermann, 1996; O’Leary and Smith, 2004; Annicchiarico et al., 2019). Well performing genotypes should display a high general mixing ability (GMA), i.e., lead to a high total mixture yield performance across several potential mixing partners, and a low variance in specific mixing ability (SMA), i.e., little or no specific interaction with individual mixing partners in that respect. In order to develop efficient breeding strategies for crop mixtures of two species, trial designs must be developed that allow precise estimation of GMA and SMA variance.

Current trial designs apply a factorial setup, combining *m* genotypes of species one and *n* genotypes of species two. With increasing numbers of genotypes of both mixing partners, factorial designs quickly result in an unfeasibly high number of experimental plots. Therefore, often specific dimensions of both crop species are used: depending on the question to be addressed either (i) factorials of equal (or similar) dimensions of *m* and *n* with a small to medium number of genotypes (Annicchiarico and Piano, 1994; Hauggaard-Nielsen and Jensen, 2001) or (ii) factorials of different dimensions for *m* and *n* (Annicchiarico, 2003; Hoppe, 2016; Starke, 2018) are employed. The former allows GMA and SMA estimations of both crop species involved, the latter emphasizes one species over the other and is comparable to the topcross designs, used in hybrid breeding. With advances in mixed modeling statistical software (such as with the GNU R packages “lme4” or “SOMMER”), analyses of largely incomplete datasets are possible (Bates et al., 2015; Covarrubias-Pazaran, 2016; R Core Team, 2019). Incomplete factorial designs have been suggested to mitigate the limitations of (i) and (ii) by expanding the numbers of m and n while maintaining a feasible number of experimental plots. Previously, they have been applied to assess GMA and SMA effects in wheat (*Triticum aestivum* L.) cultivar mixtures (Forst et al., 2019) and found recent application in genomic prediction in corn (*Zea mays* L.) hybrid breeding (Seye et al., 2020).

Another important area to advance breeding for mixed cropping focuses on exploiting information that is contained in the fraction yields of mixed crops via the application of the producer (Pr) and associate (As) concept (Wright, 1985; Goldringer et al., 1994; Annicchiarico et al., 2019). In the mixed cropping context, the Pr effect, sometimes also referred to as direct effect, is the capacity of a genotype to influence its own yield in a mixture, while the As effect is its capacity to influence the yield of its companion crop or variety (Annicchiarico et al., 2019). As laid out by Wright (1985), Forst (2018), and Sampoux et al. (2020), the Pr and As effects of a given genotype sum up to its GMA effect. It has been applied to single row experiments in breeding nurseries (Goldringer et al., 1994) or wheat cultivar mixtures (Forst, 2018), and to the mixed cropping context (Wright, 1985; Sampoux et al., 2020). Separated yield data enables either uni- or bivariate (or multivariate) analysis, i.e., joint analysis of the two (or multiple) fraction yields. In clinical psychology as well as in livestock breeding, multivariate analysis procedures have successfully been applied in situations where traits were correlated, e.g., due to pleiotropy, and yielded higher precision for QTL detection than univariate approaches (Sørensen et al., 2003; Meier et al., 2015). In mixed cropping conditions, it can be assumed that errors of measurements are generally negatively correlated between the two crops, e.g., via compensation effects. Often in mixed cropping, one of the species is at a competitive disadvantage and genotypes of the species that is very non-competitive generally express a low GMA in mixtures (Annicchiarico and Piano, 1994; Corre-Hellou et al., 2006). However, the competitive ability of genotypes is obscured when only whole mixture yield is observed (Annicchiarico et al., 2019). The assessment of fraction yields is not only important to identify these competitive abilities, but can also be applied to optimize a mixture toward a specific ratio, e.g., for feed nutrition or legume subsidies reasons, as is for example the case in Switzerland (Bundesamt für Landwirtschaft BLW, 2019). Thus, shaping community performance and composition via traits can be of interest in the breeding process. Furthermore, high genetic correlations between certain traits and mixture yield would allow indirect selection based on most important key traits for Pr and As effects.

The choice of an efficient trial design, the choice of an efficient analysis method and the assessment of yield proportions are interrelated topics. They provide the potential to be combined in an integrated approach to promote breeding for mixed cropping. While some published work focuses on the parallel genetic improvement of two species (Sampoux et al., 2020), many publications rely on the improvement of one species at a time and do not take the potential of analyzing separated yield data into account. Thus, the objectives of this study were to (i) develop a model to estimate GMA and SMA variances of binary species mixtures, and to compare different experimental designs for their usefulness in estimating these parameters, (ii) subdivide GMA into Pr and As effects in order to categorize cultivars’ influence on mixture yield and to compare the precision of a uni- versus a bivariate approach in estimating Pr, As, and error (co)variances, and (iii) establish a concept to link plant traits to biological interactions between involved species in mixture.

## Materials and Methods

To exemplify the case of a mixed crop, a hypothetic binary mixture of a legume species (pea) and a non-legume species (barley) will be used in this study.

### The GMA Model of Total Mixture Yield

Mixture yield can be expressed with the following model:

(1) $$y_{ijk}=\mu +r_{k}+G_{p_{i}}+G_{b_{j}}+S_{ij}+E_{ijk}$$

with *y*_*ijk*_ the total mixture yield of the i-th pea cultivar mixed with the j-th barley cultivar in the k-th block, μ the intercept of mixture yields, *r*_*k*_ the effect of the k-th block (replication), *G*_*p_i_*_ and *G*_*b_j_*_ the GMA effects the i-th pea cultivar and the j-th barley cultivar, respectively, *S*_*ij*_ the SMA effect, i.e., interaction, of the i-th pea cultivar with the j-th barley cultivar and *E*_*ijk*_ the error term.

**FIGURE 1:**
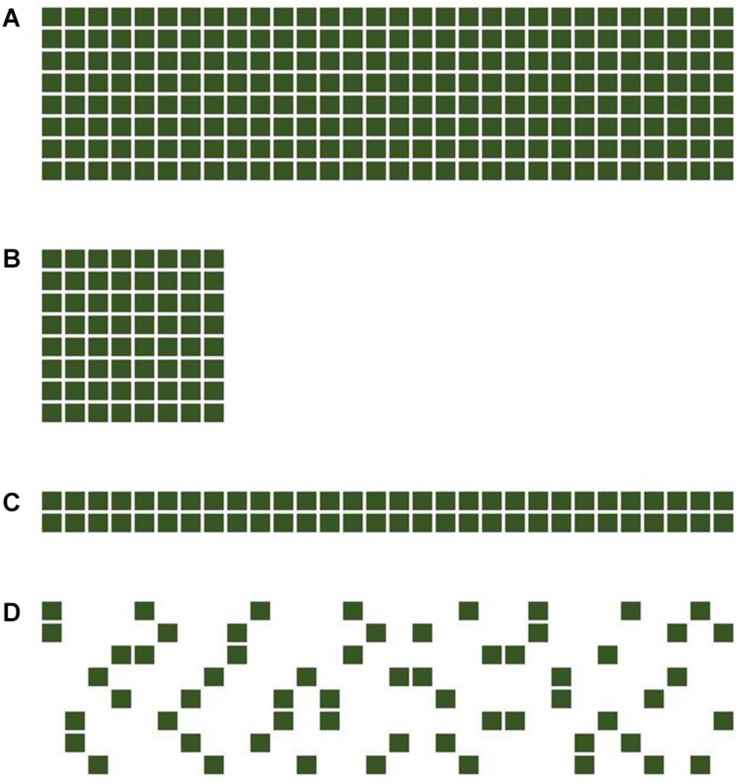
The four experimental designs used in this study, comprising three full (f) factorials with all possible pairwise combinations present and an incomplete (i) factorial with only a subset of all possible pairwise combinations present. The designs included **(A)** an 8 barleys × 30 peas full factorial (8 × 30f), **(B)** an 8 barleys × 8 peas full factorial (8 × 8f), **(C)** an 2 barleys × 30 peas full factorial (2 × 30f), and **(D)** an 8 barleys times 30 peas incomplete factorial (8 × 30i). The last three designs consume roughly the same amount of experimental resources with 64, 60, and 60 experimental plots, respectively.

This model-framework was used to compare four different trial designs (A–D), comprising three full (“f”) factorials, with all possible pairwise combinations present, and an incomplete (“i”) factorial with only a subset of all possible pairwise combinations present (Figure 1). In the following, *m* is the number of barley cultivars and *n* the number of pea cultivars, used in the design. Design A, using 240 plots per replicate (*m* = 8 and *n* = 30), is the most resource-expensive design. Designs B, C, and D were using only approximately 25% of the resources of A with, respectively, 64 (*m* = 8, *n* = 8), 60 (*m* = 8, *n* = 30), and 60 (*m* = 2, *n* = 30) plots per replicate, while sharing commonalities with design A. Designs B and C are both also full factorials (with equal and unequal dimensions of *m* and *n*) and design D shares the same size of *m* and *n* with design A while being an incomplete factorial. Design C (2 × 30f) full factorial has similarities with a top cross design, used in early stages of a hybrid breeding program. Design D (8 × 30i) was constructed using four independently randomized 8 × 8 latin squares of which only two “entries” were used for the mixtures. This ensured that (a) every barley was combined with eight pea cultivars, and every pea was combined with two different barley cultivars and (b) confounding, i.e., two or more pea cultivars sharing the same two barley cultivars was minimized.

For the comparison of the four designs, datasets with total mixture yield-data (“total yield setting”) were simulated with the following models for a “SMA present” and a “SMA absent” simulation, respectively. For the SMA absent simulation, *S*_*ij*_ was set to zero in the model in formula (1). Simulations were performed according to the following procedure, using parameter settings in the same order of magnitude as empirical values from preliminary trials (Haug et al., in preparation) to produce data that is as close to empirical data as possible. The intercept of mixture yield was set to 38.8 dt/ha. For each of the following parameters, the corresponding effects were drawn from their respective probability distributions: block effects *r*_*k*_ were drawn from a normal distribution with a mean of 0 and a variance of 2, i.e., N (0, 2). Pea GMA effects *G*_*p_i_*_ were drawn from N (3, 0), barley GMA effects *G*_*b_j_*_ were drawn from N (0, 5) and SMA effects *S*_*ij*_ were drawn from N (0, 5) for the SMA present simulation and from N (0, 0) for the SMA absent simulation, i.e., the effect size was set to zero. Errors *e*_*ijk*_ were drawn from N (0, 5). For each simulation run, effects were drawn anew. “True” values (i.e., values used to simulate data) of each effect were saved after each simulation run for later comparison with the estimated parameter values, e.g., the true GMA effects of the pea cultivars, were stored for later comparison with GMA effects estimated by the best linear unbiased predictors (BLUPs) received by the mixed model to analyze the simulated data. Design D normally would have 64 pairwise combinations. The realistic case of missing/unusable genotypes was assumed, by excluding two pea lines in our case, thus resulting in 60 pea-barley combinations. For each of the four trial designs *n* = 1,000 data sets were simulated, for the SMA present, as well as the SMA absent simulation, i.e., 8 × 1,000 simulated data-sets in total. Each dataset comprised two replicates (blocks). All simulations and subsequent analyses were done using GNU R (R Core Team, 2019). The R-code used for simulation and analysis is publically available (Haug, 2020a).

### The Pr/As Model of Fraction Yield of Each Species

Effects on separated yield data, i.e., pea and barley fraction yields, can be described by a model, containing Pr and As effects:

(2) $$y_{p_{ijk}}=\mu_{p}+r_{p_{k}}+P_{p_{i}}+A_{b_{j}}+E_{p_{ijk}}$$

(3) $$y_{b_{ijk}}=\mu_{b}+r_{b_{k}}+P_{b_{j}}+A_{p_{i}}+E_{b_{ijk}}$$

with *y*_*p_ijk_*_ the fraction yield of pea from the combination of the i-th pea cultivar with the j-th barley cultivar in the k-th block, μ_*p*_ the intercept of pea fraction yields, *P*_*p_i_*_ the effects of the k-th block on pea fraction yield, *P*_*p_i_*_ the Pr effects and *A*_*b_j_*_ the As effects of the i-th pea and the j-th barley cultivar, respectively, and *E*_*p_ijk_*_ the error for the fraction yield of the i-th pea with the j-th barley in the k-th block. Each dataset comprised two replicates (blocks). Parameters apply in analogy for barley fraction yields in formula 3. Interactions between Pr and As effects are ignored.

Since total mixture yield *y*_*ijk*_ decomposes into (*y_p_ijk__* + *y_b_ijk__*), also the other parameters can be decomposed: μ into (μ_*p*_ + μ_*b*_), *r*_*k*_ into (*r_p_k__* + *r_b_k__*), *G*_*p*_ into (*P*_*p*_ + *A*_*p*_), *G*_*b*_ into (*P*_*b*_ + *A*_*b*_), and *E*_*ijk*_ into (*E_p_ijk__* + *E_b_ijk__*). Hence, formula 1 (without *S*_*ij*_ can be rewritten as in formula 4.

(4) $$y_{p_{ijk}}+y_{b_{ijk}\;}=\;{\text{μ}}_{p}+{\text{μ}}_{b}+r_{p_{k}}+r_{b_{k}}+P_{p_{i}}+A_{p_{i}}+P_{b_{j}}+A_{b_{j}}+E_{p_{ijk}}+E_{b_{ijk}}$$

(5) $$\left[\begin{array}{c} P_{p_{i}}\\ A_{p_{i}} \end{array}\right]∼N_{2}\left(\left[\begin{array}{c} 0\\ 0 \end{array}\right],\left[\begin{array}{cc} 6.1 & -4.1\\ -4.1 & 7.5 \end{array}\right]\right)$$

(6) $$\left[\begin{array}{c} P_{b_{j}}\\ A_{b_{j}} \end{array}\right]∼N_{2}\left(\left[\begin{array}{c} 0\\ 0 \end{array}\right],\left[\begin{array}{cc} 6.1 & -2.7\\ -2.7 & 1.5 \end{array}\right]\right)$$

(7) $$\left[\begin{array}{c} P_{b_{j}}\\ A_{b_{j}} \end{array}\right]∼N_{2}\left(\left[\begin{array}{c} 0\\ 0 \end{array}\right],\left[\begin{array}{cc} 6.1 & 3.6\\ 3.6 & 8.4 \end{array}\right]\right)$$

**FIGURE 2:**
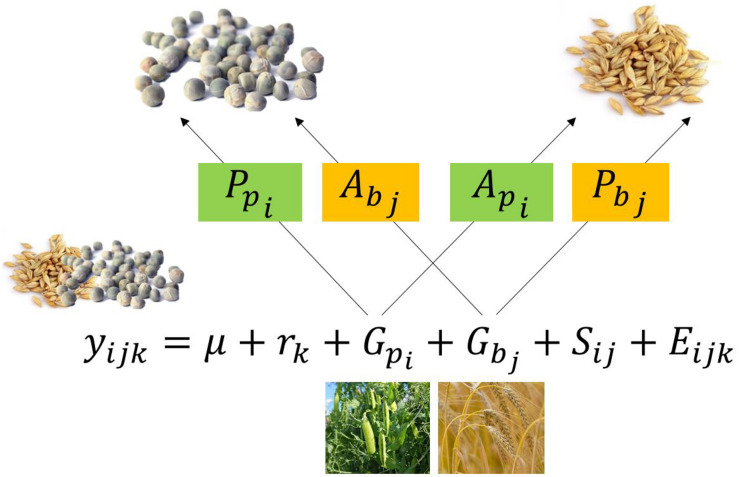
Decomposition of the general mixing ability (GMA) of pea (*G_p_i__*) in its producer (*P_p_i__*), and associate (*A_p_i__*) effects. Parameters apply in analogy for barley.

Decomposition of GMA into Pr and As effects is illustrated in Figure 2. Separated yield data (“fraction yield setting”) were simulated to compare the precision of a univariate versus a bivariate analysis approach for design D (Figure 1). As in the total yield setting, two blocks per data set were simulated. In contrast to the total yield setting, for each “plot” the separated yields of pea and barley were simulated with a mean pea yield of 18.3 dt/ha and a mean barely yield of 19.1 dt/ha, according to previously mentioned preliminary experimental data. The following settings were used: block effects *r_p_k__* and *r_b_k__* were both drawn from N (0, 2) as in the total yield setting. Pea Pr effects *P*_*p_i_*_ were drawn from N (0, 6.1) and As effects *A*_*b_j_*_ from N (0, 7.5). Barley Pr effects *P_b_j__* were drawn from N (0, 6.1) and As effects *A_p_i__* from N (0, 1.5), errors *E*_*p_ijk_*_ and *E_b_ijk__* were drawn for pea yield from N (0, 8.4) and for barley yield from N (0, 6.1). Correlations of pea Pr effects with pea As effects were set to −0.61, and correlation of barley Pr with barley As effects were set to −0.81. Within-plot error-correlations, i.e., the correlation of errors of barley yields with the errors of the corresponding pea yields in the same plot, were set to 0, −0.5, and −0.9, respectively, to create three different error-correlation scenarios. Within-plot error-correlations and Pr/As correlations were translated into co-variances. The variance-covariance matrices of Pr/As effects of pea, barley and the errors are shown in formulas 5, 6, and 7. Pr and As effects as well as errors were drawn from a distribution that follows the law of a multivariate normal distribution, using the function “mvrnorm” from the R-package “MASS” (Venables and Ripley, 2002), and the covariances shown in formulas 5–7. In total, three data sets for each of the three different error correlation settings with 1,000 simulations were created.

Simulated data from the fraction yield setting was analyzed using (i) a univariate approach with models equal to those used to simulate the data (formulas 2, 3) and (ii) a bivariate approach (formula 4) in which the two dependent variables were analyzed jointly (Covarrubias-Pazaran, 2018). In addition to the parameters estimated by the univariate approach, the bivariate approach also estimates the before mentioned covariances. Both approaches were done as mixed models where block-effects were considered as fixed and all other effects as random, assuming independent and identically distributed random variables. The uni- and the bivariate analyses were done with the “mmer” function of the R-package “SOMMER” (Covarrubias-Pazaran, 2016, 2018). Estimates of the model parameters, e.g., estimated GMA or Pr variances of pea, and BLUPs for the genetic effects, e.g., BLUPs of GMA effects, were saved for later analysis for each of the 1,000 datasets per setting. Depending on the analysis approach, each dataset yielded a different set of BLUPs for the pea and barley Pr and associate effects. For *n* = 1,000 analyses, Pearson correlations between the two sets of BLUPs and the true value were computed, Fisher-z-transformed, averaged for each coefficient and transformed back. *T*-tests between the mean correlation of the univariate and the bivariate approach within each parameter were conducted to compare the approaches for their accuracy to estimate the true effect values. The R-code used for simulation and analysis is publically available (Haug, 2020b).

### Trait versus GMA/Pr/As Analyses for the Characterization of Biological Interaction Functions (BIFs)

Beyond the purely statistical treatment of the data described above, the relationships between a fictive explanatory trait and the GMA/Pr/As variables were investigated. This explanatory trait was set into relation with the GMA, Pr, and As effects of equally fictive genotypes. Nine possible scenarios of trait-GMA, trait-Pr effect, and trait As-effect relationships were investigated, allowing the categorization of traits according to their biological interaction function in a mixed-crop plant community. Exemplary scatter plots with *n* = 100 simulated genotypes and positive, null and negative relationships between trait and GMA, trait and Pr, and trait and As effects were created to suggest a simple visual analysis of these relationships. Potential symbiotic trait functions were associated to the corresponding functions relationships.

## Results

### Incomplete Design Yields Comparable Estimates to Full Factorial Designs

Four experimental designs were compared for their ability to estimate GMA and SMA variances as well as estimating genotypic effects (BLUPs) correctly in two different simulations, a “SMA-present” and a “SMA-absent” simulation.

Over both scenarios, the precision of the estimates increased with experimental resource input (Table 1). With design A (8 × 30f), utilizing 240 experimental plots per replicate, the narrowest CIs among the four designs were received, more narrow than the ones of designs B (8 × 8f), C (2 × 8f), and D (8 × 30i), which were using only 64, 60, and 60 experimental plots per replicate, respectively (Table 1). Among the latter, only minor differences in CIs were observed (except lower reliability on GMA variance of barley in design C and on GMA variance of pea in design B in the SMA-absent simulation). Besides GMA variance of barley of design C in the SMA-present simulation and the GMA variance of pea of design B in the SMA-absent simulation, certain parameters were estimated similarly well with designs B, C, and D as with the benchmark design A. Barley GMA variance of designs B and D showed similar CIs compared to design A for both the SMA-present and the SMA-absent simulation, whereas design C, which only uses two instead of eight barley cultivars, estimated barley GMA variance less precisely (CI of ±0.43 in both scenarios). In addition, pea GMA variance of designs C and D of the SMA-absent simulation were similarly precisely estimated compared with design A, whereas design B estimated this parameter with lower precision (CI of ±0.11).

**TABLE 1:** Results on general and specific mixing ability (GMA and SMA) from simulated data for four different trial designs (A–D).

			Pea GMA		Barley GMA		SMA		Error
Simulation	Trial design^1^	Plots per replicate	Means of variance estimates (truth: 3.0)^2^	Means of correlations BLUPs vs. true effects^3^	Means of variance estimates (truth: 5.0)	Means of correlations BLUPs vs. true effects	Means of variance estimates (truth: 5.0/0.0)	Means of correlations BLUPs vs. true effects	Means of variance estimates (truth: 5.0)
SMA-present simulation	**A** (8 × 30f)	240	2.97 ± 0.06	0.88^c^	4.98 ± 0.17	0.98^c^	5.00 ± 0.05	0.77^d^	5.02 ± 0.03
	**B** (8 × 8f)	64	2.90 ± 0.13	0.89^d^	4.98 ± 0.19	0.93^b^	5.03 ± 0.10	0.74^c^	5.02 ± 0.06
	**C** (2 × 30f)	60	3.06 ± 0.12	0.68^b^	4.64 ± 0.43	– ^4^	4.85 ± 0.12	0.70^b^	5.03 ± 0.00
	**D** (8 × 30i)	60	3.01 ± 0.13	0.65^a^	5.13 ± 0.21	0.91^a^	4.95 ± 0.13	0.67^a^	5.02 ± 0.06
SMA-absent simulation	**A** (8 × 30f)	240	2.98 ± 0.05	0.95^c^	4.94 ± 0.17	0.99^c^	0.14 ± 0.01	– ^5^	4.88 ± 0.02
	**B** (8 × 8f)	64	2.90 ± 0.11	0.96^d^	4.94 ± 0.18	0.98^b^	0.27 ± 0.02	–	4.76 ± 0.04
	**C** (2 × 30f)	60	2.89 ± 0.07	0.84^b^	4.64 ± 0.43	– ^4^	0.35 ± 0.03	–	4.76 ± 0.05
	**D** (8 × 30i)	60	2.84 ± 0.07	0.83^a^	4.89 ± 0.18	0.96^a^	0.36 ± 0.03	–	4.80 ± 0.05

*The four designs employ different amounts of resources in terms of experimental plots per replicate, with design A being the benchmark. Numbers after ± denote the 95% confidence intervals for the mean of 1,000 variance estimates. Different letters following correlation values between best linear unbiased predictors (BLUPs) and true effects indicate significant differences (Tukey–HSD; *p* < 0.05).*

*^1^the different designs as described in Figure 1^2^means of 1000 estimates per design and 95% confidence intervals ^3^means of rho-z-rho transformed Pearson correlations, see “Materials and Methods,” letters a–d indicate significant differences with *p* < 0.001 ^4^correlation of only two data-points is by definition one ^5^correlations were not computed in scenario two, for the true SMA-effects all being 0 by definition.*

Besides the variation of estimates, a check of the correct estimation of the size of the parameter itself revealed that for the SMA-present simulation (Table 1), all four experimental designs accurately estimated GMA, SMA, and error variances, with all confidence intervals (CIs) of means overlapping the true values, except for the SMA variance of design C.

For the SMA-absent simulation, pea and barley GMA variances were mostly accurately estimated in the four designs with significant but small underestimations of pea GMA variance by designs C and D. SMA variances in this simulation were significantly overestimated and error variances were significantly underestimated for all four designs. Compared with the mean SMA variance of the benchmark design A of 0.14, in this simulation designs B, C, and D showed significantly higher SMA variances with 0.27, 0.35, and 0.36, respectively. Similarly, the mean error variance of design A was with 4.88 significantly higher (and thus closer to the truth of 5.0) than the error variances of designs B, C, and D, with means between 4.76 and 4.80.

When comparing the four designs for their correlation of BLUPs with the truth value, (Table 1) the correlation coefficients of benchmark design A were among the highest across all estimated BLUPs. However, correlations of BLUPs of pea GMA effects with the true effects of design B were similar compared with the benchmark design A for both simulations. For the correlations of BLUPs for pea GMA with their true values of designs C and D showed significantly lower correlation coefficients compared with design A and B for both SMA simulation models. However, this difference was less apparent in the SMA-absent simulation with correlation coefficients of 0.84 and 0.83 of designs C and D compared with 0.95 and 0.96 of designs A and B, respectively. All correlation coefficients differed significantly from each other (*p* < 0.001). For barley GMA, design B and D showed with 0.93 and 0.91 high mean correlation coefficients that were similar to the mean of design A (0.98). Correlation coefficients for SMA effects were in the range of the correlation coefficients for pea GMA effects and barley GMA effects, with values between 0.67 (design D) and 0.77 (design A).

### The Pr/As Concept Allows to Characterize Cultivars’ Contribution to Mixture Yield

**FIGURE 3:**
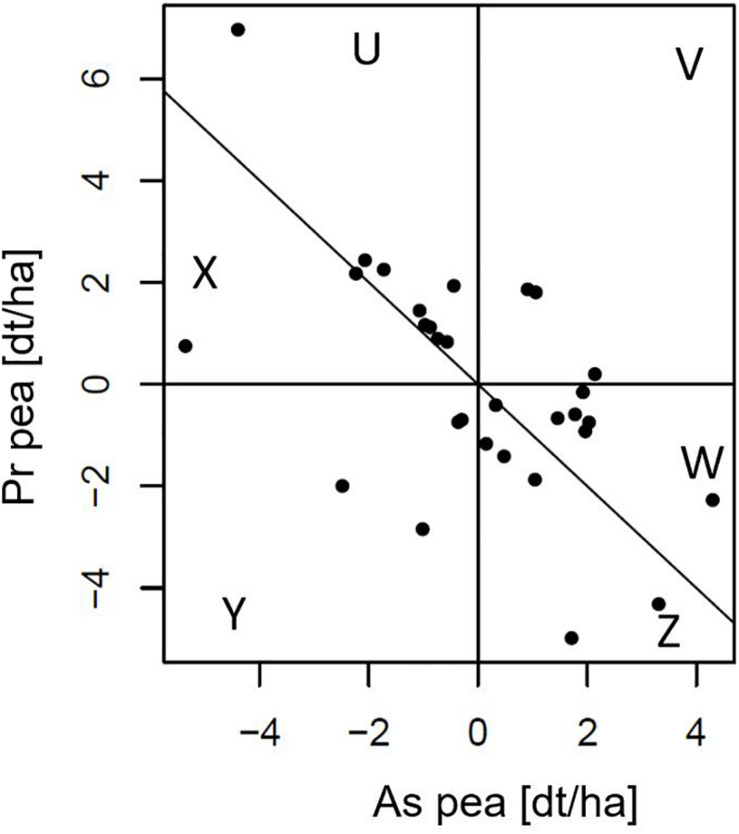
Best linear unbiased predictors of producer (Pr) and associate (As) effects of 30 pea cultivars of a bivariate analysis of simulated data in which an 8 barleys × 30 peas incomplete factorial design was used (Design C). Pr and As effects represent the yield effects in dt/ha of cultivars of a focal species (here pea) on partial yields of itself (Pr) or on the associated species (As; here barley; read “As effect of pea on barley yields”). Data taken from a randomly chosen simulated data set of the Pr/As data. The sum of the Pr and As effects of a cultivar equals its GMA effect, thus, the line with slope –1 and intercept 0 separates genotypes with positive (above) and negative (below) GMA. Genotypes can have a positive GMA by either a high Pr effect that offsets for a negative. As effect (sector U), both positive Pr and As effects (sector V) or a high As effect that offsets for a negative Pr effect (sector W; consequently opposite for sectors X, Y, and Z).

In Figure 3, thirty simulated cultivars with their Pr and As effects are shown. Pr effects range from −5.0 to +7.0 and As effects range from −5.4 to +4.3. As effect has to be read as the effect of the species (e.g., pea) on the yield (or any other trait) of its companion species (e.g., barley). Since the Pr and As effects of a cultivar sum up to its GMA, cultivars that lie on the line with slope −1 and intercept 0 have a GMA of zero, those above this line have a positive and below a negative GMA. The cultivars thus can be grouped into six sectors, U, V, W, X, Y, and Z, with U–W having positive GMA due to a high Pr effect that offsets a negative As effect (sector U), both a positive Pr and As effect (sector V) and a positive As effect that makes up for a negative Pr effect (sector W). On the other hand, cultivars below the identity line have a negative GMA with a positive Pr effect that does not compensate for a negative As effect (sector X), both negative Pr and As effects (sector Y) and a negative Pr effect which is not offset by their positive As effect (sector Z). These six sectors allow to characterize and differentiate the mixing ability of the pea cultivars.

Comparing the uni- and bivariate approach using the data from the fraction yield setting, correlation of errors of 0, −0.5, and −0.9 resulted in very similar parameter estimates (Supplementary Table 1). The “error correlation of −0.5 scenario” was used to analyze separated fraction yield data of both models in more detail. Both analysis approaches, uni- and bivariate, produced unbiased results of parameter estimates, i.e., all 95% confidence intervals of the estimates contained the true values for both approaches (see Supplementary Table 2). Estimates did not differ significantly between the univariate and the bivariate analysis approach. CIs, used as a measure for precision, differed only by 0.01 or not at all. However, only the bivariate model allows to estimate the correlation between Pr and As effects.

### Pr/As-Trait Relationships Uncover Biological Interaction Functions (BIFs) of Traits

The GMA, Pr, and As effects on total or fraction yield do not reveal the underlying biological processes or traits that influence the mixing ability. Yet, the examination of relationships between a fictive explanatory trait and Pr/As effects on fraction yield fills this lack by defining nine potential biological interaction functions (BIFs) of a given trait that underlie the GMA-trait pattern (Figure 3). This GMA-trait relationship can be positive (+), absent (0) or negative (−). However, the GMA-trait relationship is subdivided in its underpinned three possible Pr-trait/As-trait relationships. These can then be interpreted in terms of BIFs: commensalism (Pr+/As0, Pr0/As+, i.e., trait will profit only one species), mutualism (Pr+/As+, i.e., trait promotes both species), antagonism (Pr+/As−, Pr−/As+, i.e., trait promotes one species but hampers second species), neutralism (Pr0/As0, i.e., trait does not affect any of the two species), amensalism (Pr0/As−, Pr−/As0, i.e., trait is hampering only one species), and competition (Pr−/As−, i.e., trait is hampering both species). This more detailed correlations will allow to identify key traits that are important for good mixing ability for a crop and can contribute to indirect selection.

## Discussion

The overall goal of this study was to develop a novel framework for breeding for mixed cropping by (i) formulating models for mixed cropping, suggesting experimental designs and analysis methods for mixed cropping experiments, (ii) proposing extensions to the use of the Pr/As concept, and (iii) linking the latter to traits in order to uncover the biological interaction function (BIF) of traits.

### Incomplete Designs to Increase Selection Intensities

With all four designs, GMA, SMA, and error variances were overall correctly estimated, i.e., with little or no bias. As expected, the low-resource designs B, C, and D showed a slightly lower precision (i.e., higher CIs). The comparison of the three low-resource designs, incomplete factorial design (D) versus the two full factorial designs B and C, revealed similar estimations of GMA, SMA, and error variances. Design C (with two barley genotypes) does not allow a meaningful estimation of GMA variance. Thus, for an estimation of both species’ GMA variance and SMA variance, designs B or D are preferred, which is in line with the suggestion of Annicchiarico et al. (2019). Seye et al. (2020) used an incomplete factorial design for hybrid testing that was created by crossing one inbred line of one pool with one inbred line from the opposite other pool and compared to a classical topcross design where all inbred lines of pool 1 were crossed with the same line of pool 2. In the incomplete design, estimates for general combining ability (GCA) for both parents of a hybrid cannot be disentangled and are identical. Nonetheless, they emphasize, that if one only considers selection among the tested lines of the training set, the incomplete factorial (similar to design D of this study) always outperformed a topcross design (similar to design C) in terms of genetic gain, since twice the amount of genotypes (similar to barley in our case) could be tested and consequently selection intensity could be twice as high.

Correlations of true with estimated pea GMA values are lower in the incomplete design D compared with design B. In the absence of SMA, however, correlations come close to those of design B and even high-resource design A. This suggests that incomplete factorials can combine the advantages and applications of both designs with equal or similar dimensions of *m* and *n* and of designs with unequal dimensions of *m* and *n*, the latter being similar to topcross designs in hybrid breeding. At similar resource requirements, incomplete factorials allow more genotypes to be tested without a substantial loss of GMA precision and accuracy. This will allow to increase selection intensity similar to the example shown in maize hybrid breeding (Seye et al., 2020). Moreover, testing larger sets of genotypes allow to exploit larger genetic variance of a given species. Since selection gain depends on both intensity and genetic variance, incomplete designs have a great potential to increase selection gain when breeding for mixed cropping.

### Incomplete Designs for Early and Later Stages of Breeding for Mixed Cropping

Besides having been suggested for calibration of genomic prediction models for hybrid breeding (Seye et al., 2020), incomplete designs have been used to estimate GMA and SMA effects in wheat cultivar mixtures (Forst et al., 2019). The findings of Forst et al. (2019) could be applied to hybrid breeding as well as to breeding for mixed cropping: in the early development of a hybrid selection scheme for a crop, a broad range of genotypes could be tested in an incomplete diallel, similar to the one in Forst et al. (2019) that identified suitable material to form “pools” in cultivar mixtures. In mixed cropping, in early stages of breeding, where the size of the GMA variances of the two species are yet unknown and both species are of equal interest, an incomplete factorial with equal sizes of *m* and *n* would be advisable to subsequently design a breeding scheme based on the results. In later stages of both hybrid breeding (heterotic pools have been formed) and breeding for mixed cropping (focal species has been chosen), an incomplete factorial, e.g., in the form of design D, could be applied to both pools (hybrid breeding) or the focal species (mixed cropping). Only little literature has been published on actual experiments for breeding for mixed cropping. Some authors focus on the stepwise approach, first conducting a topcross design (similar to design C) to identify most promising genotypes for mixtures, followed by a full factorial to identify best combinations (similar to design B) for the development of two components of a mixture, such as species mixtures of maize (Hoppe, 2016) or common bean (Starke, 2018). The results presented in this study suggest such stepwise experiments could have been combined to a single one by the application of an incomplete design, thus speeding up the selection process. Incomplete designs can be applied to similar problems where factorial experimental designs are used, notably hybrid breeding, animal breeding.

### Pr/As Concept to Select Genotypes According to Their Species-Specific Mixing Ability

The Pr/As concept allows to cluster genotypes into groups of particular “mixing-behaviors” within positive or negative GMA. Therefore, depending on the desired proportion of fraction yield by farmers, in our example a larger ratio of pea to barley, pea genotypes can be selected from either sectors U or V of Figure 3, while pea genotypes of sector W would support a higher proportion of barley. The Pr/As concept also allows to select for GMA maximization via a regression of the Pr effects on the As effects, i.e., fit a regression to the Figure 3 dataset. For instance, a regression with a slope strictly steeper than −1 (e.g., −1.5) indicates total yield can be increased by more competitive cultivars of the focal species, thus, GMA is maximized via the selection toward higher Pr or lower As effects.

The Pr/As concept can be seen as the genetic correspondence to the replacement series as, for example, described by Wendling et al. (2017). They compared four different crop species in pairwise combinations for their biomass yield under mixed cropping. Both the Pr/As concept and replacement series describe levels of competitiveness between two species under varying competitive conditions within the mixture, conveyed by different sowing ratios in the replacement series, and by genetic differences in competitiveness in the Pr/As concept. The low and high sowing ratios of a species in the replacement series would then correspond to low (positive As effects) or high competitive (negative As effects) genotypes, respectively. Wendling et al. (2017) observed linear relationships between mixed crop species only in two out of twelve replacement series. For all other scenarios, local maxima with transgressive overyielding were identified, i.e., mixture biomass yield exceeded the pure-stand biomass yield of each species. Due to the resemblance of the two concepts, it is quite possible, that similar local maxima for mixture yield occur in a Pr/As context. In this case, instead of a linear regression, bi-, polynomial, local, or non-parametric regressions could be applied, in order to find a target interval for As values to maximize mixture yield.

Pr and As effects are correctly estimated without being biased by different levels of correlated errors. A bivariate model is provided, able to take such a correlation into account. This model is an original analysis approach and the canonical way to treat paired variables that are presenting obvious inter-dependencies (yield of two crops cultivated on the same plot). However, this bivariate approach, with our design chosen to ensure balance and avoid confounding as much as possible, did not yield an improvement in terms of precision of estimates compared with the univariate approach. This is against our expectation and suggesting literature (Sørensen et al., 2003; Meier et al., 2015) but the focus of this study was on parameter estimation, whereas strengths of bivariate approaches might rather lie in other applications, such as hypothesis testing and outcome prediction. Even though the precision of estimates could not be improved with it, the multivariate approach can be used to estimate genetic correlations between traits (Meyer, 1991), which is fundamental for the use of indirect selection methods in mixed crops as suggested by Annicchiarico et al. (2019).

### Pr/As-Trait Relationships to Shape Species Mixtures

**FIGURE 4:**
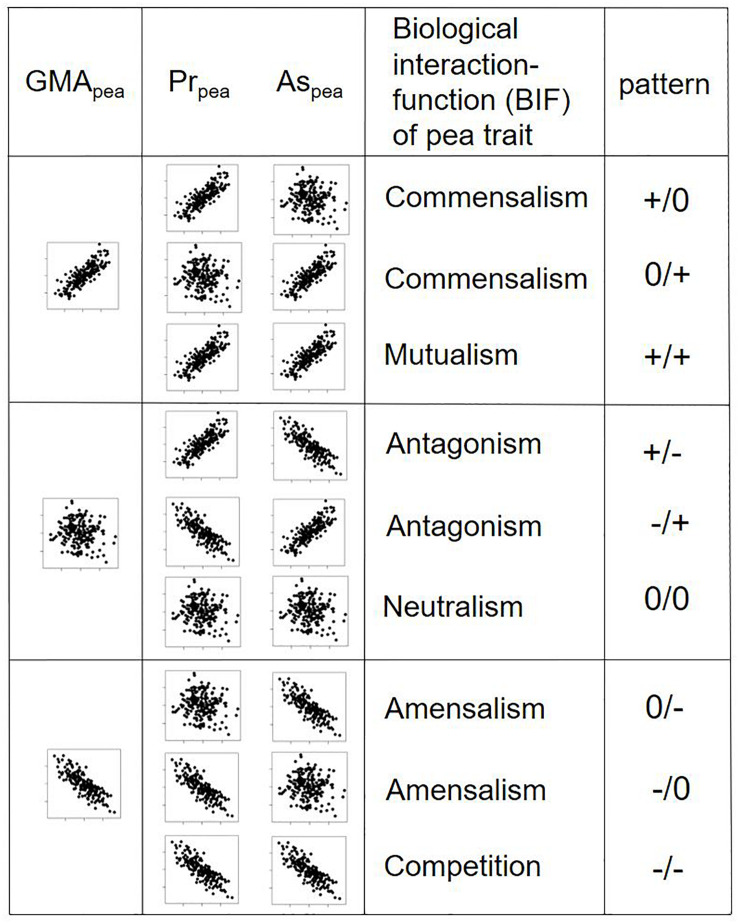
Schematic representation of pea genotypes with three potential relationships (positively correlated, uncorrelated, negatively correlated) of their GMA with a fictive explanatory trait and three potential underlying Pr- and As-trait relationships. Values of the explanatory trait lie on the *x*-axis, GMA, Pr, and As values on the *y*-axis. Pr/As-trait relationships reveal different biologic interaction functions (BIFs). The pattern describes a neutral (0), positive (+) or negative (–) influence on the species on which the trait was measured (left of the slash) or the species associated to this species (right of the slash).

The Pr/As concept can be seen as an extension of the concept of competitive effect and response (Goldberg and Fleetwood, 1987). There, a relationship or effect of trait A (e.g., early vigor), measured in species one (competitive effect) on a different trait B (e.g., yield), measured in species two (competitive response) is assumed. In the Pr/As context, however, trait A in species one (e.g., early vigor) can have an effect on a trait B that is common in both species (e.g., yield of species one and two, i.e., Pr/As effects), as visualized in Figure 4. By combining the Pr/As concept with trait measurements, the BIF of a trait can be determined. This bears the potential for further systematic investigation and categorization of trait functions in mixed cropping and community ecology, where it might serve to discover, which trait categories prevail in successful plant or other organismic communities, and shape the functioning – or non-functioning – coexistence of these. The identification of BIFs is therefore very important for breeding for crop mixture, which is not possible, if only the trait-GMA relationship (i.e., total yield) is being looked at. Nevertheless, analyzing correlations between GMA and traits can still be of interest to identify key traits that influence total mixture performance like in forage crops but not the performance of individual species.

## Conclusion

A GMA/SMA model could be applied to compare different experimental designs for their capacity to provide meaningful information for breeders engaging in mixed cropping. Based on our findings, we recommend to use an incomplete factorial design in early stages of breeding for mixed cropping, since it allows to extend the number of tested cultivars at equal levels of experimental resources. Breeding programs can be sped up by the possibility to merge otherwise stepwise full factorial experiments into one single step. The Pr/As concept applied to an incomplete factorial design was shown to be an adequate tool to optimize mixture yield. It enables (i) to select genotypes with a suitable GMA-type thus optimizing mixture composition and (ii) to identify competitive optima for yield maximization in mixed cropping. It further allows to characterize the function of traits within species mixtures by their BIF and thus gain knowledge about their role in the biological interactions between species in a plant community. For ease of comprehension, the current study does not take genotype × environment (G×E) interactions into account. Future research should address these interactions, and the models and methodology provided here can be expanded to integrate this interaction. This study provides an integrative methodological approach for the emerging field of breeding for mixed cropping of arable crops.

## Data Availability Statement

The R-codes used for the simulations and analyses supporting the conclusions of this article were made available by the authors under (see also Haug, 2020a,b).

## Author Contributions

BH wrote the manuscript. The simulation experiments were designed and its statistical framework was established by all authors. IG, JE, TMH, EF, MM, PH, and BH developed the incomplete design idea. TF, TMH, and BH wrote the R-scripts for the analyses. All authors reviewed the final manuscript.

## Conflict of Interest

The authors declare that the research was conducted in the absence of any commercial or financial relationships that could be construed as a potential conflict of interest.
