# Supplementary material for: Advances in Breeding for Mixed Cropping – Incomplete Factorials and the Producer/Associate Concept
Source: Front Plant Sci. 2021 Jan 11;11:620400. doi: 10.3389/fpls.2020.620400 (PMC7829252; doi:10.3389/fpls.2020.620400)
Supplement: Supplementary file 1 [file Table_1.DOCX]

**Supplementary table 1**. Comparison of two different analysis-approaches of fraction-yields of a mixed cropping experiment (experimental design D 8x30i, see Figure 1), tested with 1000 simulated data-sets. SMA was assumed to be 0 and correlation of errors of pea and barley fraction yield in a plot was assumed to be either -0.9, -0.5 or 0. The first approach estimates the parameters with a univariate (formulas 2 and 3), the second with a bivariate model (formula 4).
