# Supplementary material for: Advances in Breeding for Mixed Cropping – Incomplete Factorials and the Producer/Associate Concept
Source: Front Plant Sci. 2021 Jan 11;11:620400. doi: 10.3389/fpls.2020.620400 (PMC7829252; doi:10.3389/fpls.2020.620400)

**Supplementary table 2**. Confidence intervals (CIs) and truth vs. BLUP correlation of the “error correlation -0.5 scenario” (supplementary table 1). The uni- and bivariate models are described in formulas 2, 3 and 4. Since producer and associate effects of a genotype sum up to its GMA, for completeness also the GMA variance is given in brackets. The ± sign indicates the 95% confidence intervals for the variance estimates.


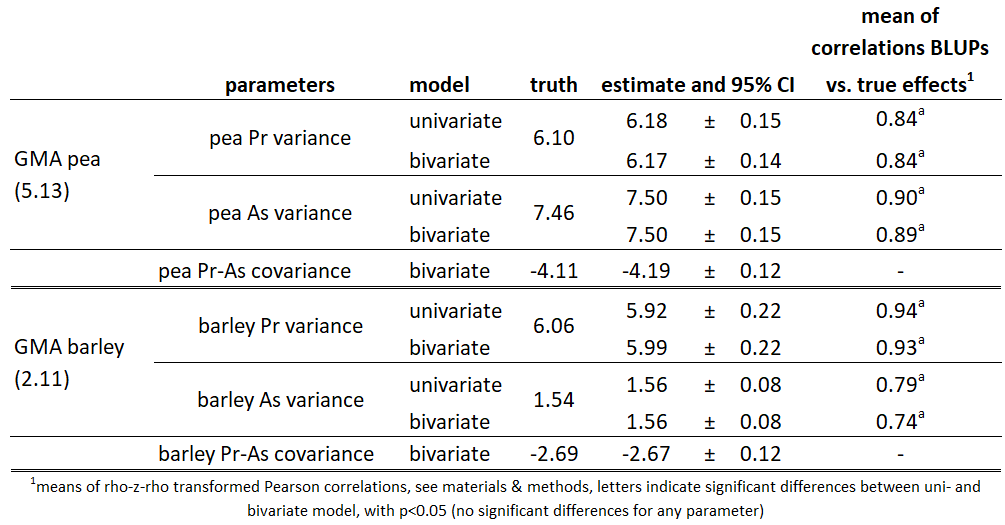

Supplement: Supplementary file 2 [file Table_2.DOCX]
